# Effects of the Family Status of Osteopathic Medical Students on Their Perceived Level of Stress

**DOI:** 10.7759/cureus.62764

**Published:** 2024-06-20

**Authors:** Mikhail Volokitin, Tommaso Meregalli, Ricki Ehrenfeld, Denise K Burns

**Affiliations:** 1 Osteopathic Manipulative Medicine, Touro College of Osteopathic Medicine, New York City, USA

**Keywords:** medical school, family obligations, stress, medical students, marriage

## Abstract

Osteopathic medical students face an exceptional stress-inducing 4-year period of their lives while in medical school. Students who might have an interest in entering marriage during medical school may hesitate to commit, perceiving marriage as a possible additional stressor to the already complex and vast medical curriculum. This investigation aimed to understand the effects of marital status on osteopathic medical student stress levels. An online survey was conducted, and 100 responses were recorded and analyzed. To measure stress levels, the Perceived Stress Scale was utilized. Raw stress scores were compared utilizing t^2^ analysis while stress level categories, such as low stress, moderate stress, and high stress, were compared using χ^2^ analysis. The findings show that there is no significant difference between osteopathic medical students who are married and those who are not when comparing for both, raw stress score (P=0.092), and stress score level (P=0.186). These results conclude that marriage does not act as an exacerbator or alleviator for osteopathic medical student stress levels.

## Introduction

Medical school has been widely acknowledged as a challenging and stress-inducing portion of physician training. During medical education, student physicians face numerous challenges and display elevated stress levels when compared to their peers in the general population [1]. The causes of such heightened stress have been previously investigated, listing competition, rather than cooperation between students, burnout, and financial hardships, as contributing factors [2]. Furthermore, the effects of exposure to such emotional disturbance have been understood to result in negative effects on the cognitive functioning and learning of medical students [3]. Given the multitude of stressors medical students face on their path to becoming physicians, it is understandable how students might hesitate to add additional potential stressors to their lives, such as marriage. Previous studies have analyzed the effects of marital status as a potential stressor and have found that being married can decrease stress levels in medical school [4], additionally, stress levels are attenuated with perceived spousal support [5]. The previous literature on the topic is limited and, due to the lack of recent investigations, the findings might have changed over time. Different factors about marriage have drastically changed over the past half-century. Over the past 40 years, the rate of marriage has seen a 12% increase, from 49% in 1982 to 61 % in 2022 [6]. In 1982, the median age of marriage was in the range of 23-25 years of age, comparatively in 2022 the median age of marriage was in the range of 28-30 years of age [6] the 5-year shift was seen when comparing the median age of marriage from 1982 to 2022 might affect stress levels. Given the fact that the median age of medical students was recently found to be 24 years of age [7], this implies married medical students would either be entering marriage at a significantly younger age when compared to their peers in the general population or entering medical school at a significantly older age than their peers which may affect stress levels [6]. Previous studies have been inconclusive in assessing whether familial obligations were a protective or predisposing factor to stress [8]. Previous literature on the topic did not assess the stress levels of those who were unmarried with kids [4-5]. Since the rate of women having children outside of marriage has increased from 18% in 1980 to 40% in 2021, we decided to assess the stress levels of osteopathic medical students who are unmarried with children [9]. Similarly, familial obligations were not previously included when assessing stress levels [4-5]. This study aims to investigate whether marriage and familial obligations affect stress levels of osteopathic medical students. Furthermore, we want to broaden the scope of our investigation to analyze the stress levels of students in the following categories: married with children, married without children, unmarried with children, and familial special caregivers. We hypothesize that marriage or familial status would not exacerbate or alleviate stress levels of medical students.

## Materials and methods

The participants of this cross-sectional survey study (n=100) were osteopathic medical students, 250 osteopathic medical students were initially invited to participate and 100 completed the survey. The sample size of 100 was determined as an arbitrary sample size and convenience sampling was used. The participants were recruited to complete a demographic survey followed by an assessment of their current stress level. The questionnaire consisted of two sections and a total of 14 questions. The respondents were given 30 days to respond to the survey. The data was collected via “SurveyMonkey”, a user-friendly survey generator granting anonymity, confidentiality, and secure data storage. Previous research has described SurveyMonkey as a cloud-based software survey service company that was established in 1999 [10]. As a survey generator, the website also provides a suite of paid back-end programs that incorporate data analysis, selection of the samples, elimination of bias, and visual tools to represent data [10]. SurveyMonkey was also found to be utilized by companies that are interested in data analysis, such as Facebook, Samsung, and Kraft Foods [10]. Most of the participants identified as females (65%), with males constituting a minority of participants (35%). The most common age range of participants was between 25-34 (70%), followed by 18-24 (23%), and 35- 44 (7%). The participant ethnic breakdown was as follows: White (43%), Hispanic (18 %), Asian (17%), unspecified (13%), and Black (9%). On familial status the participants self-identified as unmarried (65%), married without kids (17%), married with kids (16%), familial special caregiver (1%), and unmarried with kids (1%). Participant stress levels were assessed utilizing the “Perceived Stress Scale”, a widely used psychological instrument for measuring the perception of stress [11]. The Perceived Stress Scale is known to be one of the most utilized assessments for measuring psychological stress [12]. As a self-reported questionnaire, it was designed to measure “the degree to which individuals appraise situations in their lives as stressful” [11]. The Perceived Stress Scale aims to evaluate how individuals believe their lives have been unpredictable, uncontrollable, and overloaded over the past month [12]. The questions of the Perceived Stress Scale are general and do not focus on specific events or experiences [12]. The “Perceived Stress Scale” consists of a series of 10 questions about the feelings and thoughts of participants over the past month [11]. The respondents qualified their responses on a scale from 0 to 4 according to the following criteria: 0 - never, 1 - almost never, 2 - sometimes, 3 - fairly often, and 4 - very often [11]. The scores for each of the 10 questions were then added for each participant and stress levels were identified using the following criteria. Scores ranging from 0 to 13 were considered low stress. Scores ranging from 14 to 26 were considered moderate stress scores. Scores ranging from 27 to 40 were considered as high perceived stress. Once the responses were obtained the data was analyzed. The raw stress scores were compared using a two-tailed t-test. The stress levels, such as moderate stress or high stress, were compared using a chi-squared test.

## Results

The results of this study show that there is no statistically significant difference when comparing the stress levels of married medical students and unmarried medical students (Tables 1-2). The participants of this study were 100, of those 65 identified as female and 35 identified as male.

**Table 1:** T-test results comparing raw stress scores for married and unmarried osteopathic medical students.

Familial Status	N	Mean	SD	dF	P value	Decision
Married	33	22.71	3.07	97	0.092	Fail to Reject
Unmarried	67	23.91	3.62			

**Table 2:** χ2 test results comparing stress score levels for married and unmarried osteopathic medical students.

N	dF	χ^2^	P value	Decision
99	1	1.75	0.186	Fail to Reject

The raw scores from the “Perceived Stress Scale” were compared between married medical students and unmarried medical students, yielding no statistically significant difference (P=0.09, Table 1). The overwhelming majority (82%) of participants were found to be experiencing moderate stress levels, followed by participants experiencing high stress levels (18%). No participants were found to be experiencing low-stress levels. When comparing stress levels, as in moderate stress or high perceived stress, between married and unmarried osteopathic medical students no significant difference was found (p=0.18, Table 2). These findings fail to reject the hypothesis that marriage or familial status would not exacerbate or alleviate the stress levels of medical students. The study aimed to additionally analyze the effects of stress on medical students who had family commitments other than being married, such as those unmarried with kids and familial special caregivers. Given the low response (1%) for each of these categories, we were unable to further investigate the stress levels in these specific categories.

## Discussion

This study aimed to determine the role that marriage and familial obligations play in affecting the perceived stress levels of osteopathic medical students. The findings show that there is no significant difference between osteopathic medical students who are married and those who are not when comparing for both, raw stress score (Table 1, P= 0.092), and stress score level (Table 2, P=0.186). This finding is of particular interest as it is in contrast with previous literature on marriage and the effects of said commitment on medical student stress levels. The previous literature on the topic is limited and has yielded different results. Some previous studies show that marriage appeared to significantly improve medical student stress levels, [4] while other studies show that marriage appeared to significantly exacerbate medical students’ stress levels [5]. A plausible explanation for the disparity in the findings would be that over the past 40 years, the perception of stress about marital status in osteopathic medical students has varied due to the nature of the stressors changing over time, the increase in resources now available to students to improve stress management, and the shift in the median age of marriage seen in the general population. Medical education has also evolved over the past 40 years, with many institutions now providing access to mental well-being resources such as counseling and guided wellness programs [13], possibly resulting in the reduction of stress levels when compared to the findings of Katz et al. Medical students entering marriage earlier than the general population or students entering medical school later compared to their peers [6], might explain the increase in stress levels compared to the Coombs and Fawzy. The limitations of this study include students being from the same institution. The sample size (n=100) also limited data collection. It is suggested that larger, multi-institutional, and longitudinal studies be carried out to gain a deeper perspective on perceived stress levels among nontraditional students. The overall high level of stress in medical students can be attributed to the normal stressors of daily life as well as to the additional stress of course workload, lack of leisure time, material to be learned, and frequent academic examinations in a competitive environment [14]. A significant interaction effect was found indicating that school connectedness moderated the relationship between family obligations and emotional distress. Specifically, for students with low to moderate levels of family obligations, a stronger sense of school connectedness was associated with lower emotional distress. The buffering effect of school connectedness was weakened as the level of family obligations increased and completely disappeared for students who experienced high levels of family obligations [15]. In future studies, it would be interesting to investigate the differences in stress levels of medical students compared to non-medical students, as well as assess what role school connectedness would play in those who are married and how it would affect their stress levels.

## Conclusions

This study concluded that marital status does not appear to significantly affect osteopathic medical student stress levels. Previous studies have found that being married can decrease stress levels in medical students, and that stress levels are attenuated with perceived spousal support. We believe that such discrepancy in findings could potentially be attributed to our study being conducted in 2023, as opposed to the previous literature being written several decades ago. Over the years, we hypothesize that stress levels varied due to the nature of the stressors changing over time, the increase in resources now available to students to improve stress management, and the shift in the median age of marriage seen in the general population. Based on these findings medical students should feel comfortable with entering marriage during medical school if their main cause of hesitancy is fear of exacerbation of stress levels driven by marital commitment.

## Appendices

Effects of family status of osteopathic medical students on their perceived level of stress

Demographics questionnaire

 1. What is your age?

18-24 years old

25-34 years old

35-44 years old

45-54 years old

Over 55 years old

 2. What is your gender?

Male

Female

Other

3. What is your ethnicity?

White/Caucasian

Hispanic/Latino

Black/African American

Native American

Asian/Pacific Islander

Other

4. Of the following categories please select one, or more, that apply to you.

Married

Married with kids

Not married with kids

Familial special care caregiver (such as caring for a loved one: parent, spouse, child)

Not married without kids.

Stress level assessment

For each question please choose from the following alternatives:

 0 - never 1 - almost never 2 - sometimes 3 - fairly often 4 - very often

5. In the last month, how often have you been upset because of something that

 happened unexpectedly?

0 - never

1 - almost never

2 - sometimes

3 - fairly often

4 - very often

6. In the last month, how often have you felt that you were unable to control the

important things in your life?

0 - never

1 - almost never

2 - sometimes

3 - fairly often

4 - very often

7. In the last month, how often have you felt nervous and stressed?

0 - never

1 - almost never

2 - sometimes

3 - fairly often

4 - very often

8. In the last month, how often have you felt confident about your ability to handle your personal problems?

0 - never

1 - almost never

2 - sometimes

3 - fairly often

4 - very often

9. In the last month, how often have you felt that things were going your way?

0 - never

1 - almost never

2 - sometimes

3 - fairly often

4 - very often

10. In the last month, how often have you found that you could not cope with all the things that you had to do?

0 - never

1 - almost never

2 - sometimes

3 - fairly often

4 - very often

11. In the last month, how often have you been able to control irritations in your life?

0 - never

1 - almost never

2 - sometimes

3 - fairly often

4 - very often

12. In the last month, how often have you felt that you were on top of things?

0 - never

1 - almost never

2 - sometimes

3 - fairly often

4 - very often

13. In the last month, how often have you been angered because of things that happened that were outside of your control?

0 - never

1 - almost never

2 - sometimes

3 - fairly often

4 - very often

14. In the last month, how often have you felt difficulties were piling up so high that you could not overcome them?

0 - never

1 - almost never

2 - sometimes

3 - fairly often

4 - very often
